# Co-designing a low-intensity psychological therapy for fear of recurrence in psychosis using translational learning from fear of recurrence in oncology: protocol for intervention development for future testing in a feasibility study

**DOI:** 10.1136/bmjopen-2024-090566

**Published:** 2024-12-27

**Authors:** Stephanie Allan, Fiona Sinclair, Marta Correia, Ioanna Fragkandrea-Nixon, Alie Phiri, Gareth Jones, David Thomson, Francis Yanga, George Brown, Mark McCann, Sharon Anne Simpson, Jonathan Evans, Katie Robb, Andrew Gumley

**Affiliations:** 1 University of Glasgow, Glasgow, UK; 2 NHS Greater Glasgow and Clyde, Glasgow, UK; 3 Beatson Cancer Charity, Glasgow, UK; 4 Beatson West of Scotland Cancer Centre, Glasgow, UK; 5 Strathclyde Business School, Glasgow, UK; 6 School of Health & Wellbeing, University of Glasgow, Glasgow, UK

**Keywords:** Schizophrenia & psychotic disorders, Psychosocial Intervention, QUALITATIVE RESEARCH

## Abstract

**Abstract:**

**Introduction:**

Fear of recurrence is a transdiagnostic problem experienced by people with psychosis, which is associated with anxiety, depression and risk of future relapse events. Despite this, there is a lack of available psychological interventions for fear of recurrence, and psychological therapies for schizophrenia are often poorly implemented in general. However, low-intensity psychological therapy is available for people who experience fear of recurrence in the context of cancer, which means there is an opportunity to learn what has worked in a well-implemented psychological therapy to see if any learning can be adapted for schizophrenia care. This article describes the design, methods and expected data collection of development, acceptab***i***lity, feasibility, a***n***d preliminary outcome signals for a copro***d***uced low-intensity psycholo***g***ical intervention targeting fear ***o***f relapse in people with schizophrenia (INDIGO), which aims to develop an acceptable psychological intervention for fear of recurrence.

**Methods and analysis:**

INDIGO will use a mixed-methods approach to co-design and deliver a model and treatment pathway for a psychological intervention for people diagnosed with schizophrenia who experience fear of recurrence. The study will consist of four stages. First, in-depth interviews with mental health staff and people diagnosed with schizophrenia (with a further social network mapping task for patient participants only) to develop the intervention. Second, in-depth interviews with people who have accessed the Glasgow Fear of Recurrence service and oncology staff will be conducted to inform further development of the intervention. Third, co-design workshops will be held with people diagnosed with schizophrenia and mental health staff to co-design intervention content and the treatment pathway. Finally, people diagnosed with schizophrenia will be presented with an intervention prototype and invited to complete ‘think-aloud’ interviews to gather further feedback so adaptations can be implemented.

**Ethics and dissemination:**

The INDIGO study received ethical approval from East Midlands—Nottingham 2 Research Ethics Committee (24/EM/0124). The study received independent peer review prior to funding. This co-design study is expected to lead to a future feasibility study and, if indicated, a randomised controlled trial.

**STRENGTHS AND LIMITATIONS OF THIS STUDY**

- Engagement with people living with psychosis throughout the project will ensure data are analysed in a way that ensures its relevance to intervention development.
- Study participation is limited to participants who are fluent in the English language.
- Study participation is limited to a single geographic area.

## Introduction

 Schizophrenia is a serious mental health problem and is considered one of the most disabling conditions.^1^ A driver of emerging disability is psychotic recurrence, which is when people experience a return or an increase in psychotic symptoms. Maintenance treatment with antipsychotics has the best evidence for recurrence prevention^2^ but does not prevent recurrence entirely^3^ meaning there is a need to identify adjunctive treatment targets for recurrence prevention which can be used alongside maintenance medication. Fear of recurrence is an independent predictor of recurrence events^4^ which suggests it could be a modifiable treatment target. Additionally, while only powered to detect intervention feasibility, a single randomised controlled trial (RCT)^5^ demonstrated a reduction in fear of recurrence indicating again that it may be modifiable.

Many people living with schizophrenia do not get access to existing evidence-based care^6^ because interventions are often poorly implemented into routine care. Known as the evidence to practice gap,^7^ this describes the tendency for many interventions that have proven effective in RCTs not becoming available to patients in the ‘real world’ of clinical practice. In the case of psychosis, medium effect sizes have been observed for talking therapies.^8^ Still, they are often poorly implemented because they are high intensity which means they can be long (over 20 sessions) and are delivered by clinical psychologists who have limited capacity to deliver interventions at this scale.^9^ Before planning the development of new interventions, it is important to recognise the significant implementation problems of the existing evidence base so that implementation can be planned from the outset. There also appears to be a problem to evidence gap, where issues such as fear of recurrence do not have evidence-based treatments at all.

Psychosis care seems to suffer from a ‘double gap problem’ because the evidence to practice gap and the problem to evidence gap occur simultaneously. To address this, there is a need to develop interventions that not only target neglected clinical problems but are also designed to be implementable at scale such as having fewer sessions for mild to moderate presentations rather than assuming a high-intensity model for all problems experienced by people diagnosed with schizophrenia. People diagnosed with schizophrenia spectrum conditions have been shown to engage in low-intensity interventions which are delivered in sessions of 10 or less.^10^ This is important because the long length of traditional psychological therapies combined with a lack of capacity have created significant barriers to the availability of talking interventions for schizophrenia. An approach recommended by the Medical Research Council (MRC) / National Institute for Health and Care Research (NIHR) framework for the development and evaluation of complex interventions is ‘co-design’ where the expertise of patients and mental health staff is incorporated into intervention design to enhance the usability and acceptability of interventions.^11 12^

A further important option for developing implementable interventions is to see what has worked well in other areas of healthcare such as cancer care. A stepped care model acknowledges that not all service users require the same level of intervention. While some people experiencing severe fear of recurrence require individual sessions with a clinical psychologist, fear of recurrence can be reduced by accessing lower-intensity interventions.^13^ Within Glasgow, a service is available to patients via a third-sector organisation and has been shown to reduce fear of recurrence in survivors of breast cancer.^14^ The service has now expanded and is offered to survivors of other types of cancer. It offers patients six sessions and is based on acceptance and commitment therapy. Given that psychological interventions can be poorly implemented in psychosis, there is merit in taking a case study approach to learn about what has worked well in a service which is well implemented and while different from mental healthcare still faces many similar issues such as a lack of clinical staff time. There is a need to use exploratory methods to understand how this intervention has been implemented successfully from the point of view of staff and service users.

The cognitive interpersonal model of communication between patients and their social networks including healthcare professionals, and loved ones posits that fear of recurrence may be sustained by communication about psychosis-related phenomena such as symptoms and worries about symptoms between patients and others in their social network.^15^ Assessing social networks in psychosis is important because variations in social networks are associated with important outcomes linked to recurrence prevention such as social functioning or hospitalisation^16^ and may influence behaviours and attitudes towards problems such as worrying about relapse by providing social support.^17^ Interventions may be enhanced by incorporating network outreach so that patients are supported by those within their network^18^ but it is currently unknown to what extent this approach may be feasible within fear of recurrence in psychosis.

The proposed mixed-methods study forms the preparatory work for a feasibility study of a low-intensity intervention for fear of recurrence in psychosis and aims to understand what underpins the successful implementation of an existing cancer intervention to understand what has worked well and what could be innovated into psychosis research. Furthermore, incorporating a social networking task will expand on knowledge about how fear of recurrence is maintained and will enhance intervention development.

Data collection for this study will take place between July 2024 and July 2025. The intervention produced will be evaluated in a subsequent feasibility study with appropriate efficacy testing using single case experimental design methodology. Given the lack of standardised guidance of reporting intervention co-design protocols, we have adhered to a format used by Williamson and colleagues^19^ to ensure consistency and utility for future researchers.

The aim of this study is to develop a co-produced intervention by:

1. Using exploratory qualitative methods to understand fear of recurrence from the perspective of patients and mental health staff, and to find out their ideas about what a low-intensity intervention should include.
2. Using exploratory qualitative methods to understand the successful implementation of a local fear of recurrence in oncology service.
3. Using co-design workshops to develop a low-intensify intervention in collaboration with people who access mental health services for schizophrenia and mental health staff.
4. Evaluate the intervention prototype from the point of view of people diagnosed with schizophrenia using think aloud methodology.

## Methods

We will apply a mixed-methods approach to co-design to produce an intervention programme theory and intervention components.

### Patient and public involvement (PPI)

Involvement from a PPI group of four people who have experienced schizophrenia spectrum conditions informed the development of this protocol including deciding the initial research topic and focus, the generation of participant information sheets, informed consent processes, development of interview schedules and choice of psychological model. Throughout the co-design process, we will work with a team of four PPI colleagues to review, analyse and interpret data.

A steering group will guide project decision-making from procedural and policy perspectives to maximise project impact with four members coming from clinical psychology, oncology and co-design backgrounds. PPI colleagues will be invited to attend and input into all steering group meetings to ensure PPI feedback is embedded throughout steering group meetings.

### Co-design participants

Participants will include patients with a diagnosis of schizophrenia spectrum conditions, mental health staff who support people with psychosis, people who have accessed a low-intensity intervention service for fear of recurrence in cancer and oncology staff.

Throughout all four studies, we will use purposive sampling to try to ensure that the sample represents socioeconomic and ethnic diversity. We will draw attention to this strategy when we advertise the study within services.

### Inclusion criteria

#### Study 1

People with a schizophrenia spectrum condition diagnosis will be able to take part if they are: (1) 16 years of age and over, (2) not currently having an acute mental health crisis and (3) fluent in English.

Mental health staff will be able to take part if they are fluent in English and able to give informed consent (written or verbal options for all studies).

#### Study 2

People (16 years of age and over) who have accessed the low-intensity service provided by the Beatson Cancer Charity for fear of recurrence will be able to take part if they are fluent in English and are able to give informed consent.

Oncology staff who work in local National Health Service (NHS) oncology services will be able to take part if they are fluent in English and are able to give informed consent.

#### Study 3

People with a schizophrenia spectrum condition diagnosis will be able to take part if they are: (1) 16 years of age or over, (2) not currently having an acute mental health crisis and are able to give informed consent and (3) fluent in English.

Mental health staff will be able to take part if they are fluent in English and able to give informed consent.

#### Study 4

People with a schizophrenia spectrum condition diagnosis will be able to take part if they are: (1) 16 years of age or over, (2) not currently having an acute mental health crisis and are able to give informed consent and (3) fluent in English.

People living with schizophrenia can experience differing levels of symptomatology even when not in a state of crisis. We will purposively sample to ensure participants in Study 4 represent those living with differing levels of positive, negative and cognitive symptoms. We will confirm and quantify this by completing a short clinical assessment using the Clinical Global Impressions Scale.^20^

Expected participant numbers for each group and at each stage of the co-design process are outlined in table 1.

**Table 1:** Expected participant numbers for each group across studies

	Planned N	
	Service users	Staff
*Stage of the study*		
Study 1 (interviews)	7–10	7–10
Study 2 (interview)	7–10	7–10
Study 3 (workshop co-design activities)	10	10
Study 4 (user testing interviews)	5–10	0

### Procedure

We will collect data across four studies to inform the development of an intervention programme theory. All interview schedules and workshop guides have been co-produced with PPI colleagues.

#### Study 1

In this stage, we will carry out parallel in-depth one-to-one semistructured interviews with people diagnosed with schizophrenia, and mental health staff to learn about their experiences of fear of recurrence and what they believe should be included in a low-intensity intervention for fear of recurrence. We will also quantify levels of fear of recurrence in patient participants using the Fear of Reassurance Scale^21^ and collect demographic information before conducting the interviews. The interviews will follow these schedules:

- Online supplemental appendix A: Study 1—Patient Interview Schedule.
- Online supplemental appendix B: Study 1—Staff Interview Schedule.

For patient participants only, we will invite them to complete a structured social network interview to understand more about who people do and do not discuss fear of recurrence with and why this might be. Social network analysis makes distinctions between structural characteristics (eg, network size), compositional characteristics (eg, relationship type) and interactional characteristics (eg, frequency of contact). The social networking task interview schedule has been designed to highlight these network characteristics:

- Online supplemental appendix C: Study 1—Social Network Mapping Task.

#### Study 2

In this study, we will carry out in-depth one-to-one semistructured interviews with people who have accessed a well-implemented low-intensity intervention service for fear of recurrence in cancer, and staff working in local NHS oncology services. We will use this space to understand what has worked well from the point of view of service users, and staff who refer to this service and to find out if they have any ideas for improvement. The interviews will follow these topic guides:

- Online supplemental appendix D: Study 2—Service User Interview Schedule.
- Online supplemental appendix E: Study 2—Staff Interview Schedule.

#### Study 3

While the first two studies will gather extensive information on factors which are relevant for intervention development, the results will need to be compiled into an appropriate programme theory. Study 3 will use exploratory co-design methods to understand what a successful preliminary programme theory would look like from the point of view of patients and mental health staff. To do this, Study 3 will gather detailed co-design data from patients who have experienced fear of relapse, and mental health staff who support them, soliciting their expertise on what an acceptable and usable intervention for fear of relapse would look like. In summary, co-design in the case of Study 3 aims to understand the experience of the intended target audience while also incorporating theory, existing evidence and qualitative work gathered in earlier studies.

The data generated from Study 1 and Study 2 will be combined with existing research evidence and collated. This information will be presented to participants in separate workshops for patients and staff who will be invited to take part in workshop activities to sort and prioritise the information. The workshops will be facilitated by researchers who will take notes and offer to record voice notes of participant reflections. More specifically, the workshop activities are designed to gather stakeholder views on three key thematic areas:

### Intervention content

Aim: To discover what content should be in a low-intensity intervention for Fear of Relapse

- Researchers present themes from WP1 as tags on the wall.
- Blank tags are provided so people can write down missing things.
- Participants rate themes as ‘keep,’ ‘lose’ or ‘change’ using colour-coded post-it notes. These will be indicated by sections on the wall.

### Intervention delivery

- Researchers will present A4 paper sheets with blank intervention session mock-ups.
- Participants formulate ideas or write descriptions of what should be included in sessions, and how the information could be presented during the intervention.

### Journey mapping

Participants will be invited to map out what a current user journey looks like for people who experience fear of relapse.

- Researchers present an editable map with locations people with psychosis may find themselves: first episode, hospital, Community Psychiatric Nurse (CPN) appointments and time between appointments.
- Participants can indicate where they think people would want to engage in brief therapy.

The workshops will be held in an accessible university building. Following PPI feedback, we have an option for participants to take part in one-on-one design activities following the same format as the workshops for people who do not feel comfortable in groups. Following the collection of the detailed stakeholder feedback gathered during co-design sessions, we will work with PPI colleagues to co-design a programme theory. To ensure consistency, the workshops or one-on-one design activities will follow these schedules including warm-up and cool-down activities:

- Online supplemental appendix F: Study 3—Patient Co-Design Workshop Schedule.
- Online supplemental appendix G: Study 3—Staff Co-Design Workshop Schedule.

#### Study 4

Co-design methodologies used in Study 3 will result in the development of an initial programme theory, which will encompass a shared understanding of the inputs or components of an intervention, how an intervention is expected to work and what its processes of change are and what the intermediate outcomes and longer-term impact will be. Intervention design will be enhanced by further gathering specific feedback from people living with schizophrenia. Think Aloud Methodology^22^ is a technique used to gather data on what a person is thinking. Participants are asked to do a task, in this case viewing an intervention prototype, and are instructed to verbalise their thoughts and feelings while they perform the task. Think-aloud interviews are frequently used to gather feedback about interventions where there is a finalised example such as a mobile phone app.^23^ However, think aloud methodology has also been applied when participants have been supplied with information and a diagrammatic example of the programme theory which highlights what components an intervention has, in addition to describing how the intervention is expected to create change.^24^ This is an important option in early-stage work of interventions where it would not be ethically or morally acceptable to give a realistic version of the intervention because it may be like a therapy session.

During Study 4, we will present a draft programme theory (a plan for the intervention and how it might work) to people diagnosed with schizophrenia and invite them to take part in think-aloud interviews to gather feedback on how the intervention can be improved. This will follow the following interview schedule:

- Online supplemental appendix H: Study 4—Think Aloud Schedule.

### Data analysis

#### Study 1 and Study 2

Data from the one-on-one interviews will be analysed using framework analysis.^25^ Framework analysis is suited for this project because it allows for a combination of deductive and inductive coding, it works well in multidisciplinary research^26^ and it assists in comparing across datasets due to its matrix output. The deductive coding framework will use relevant facets from the core elements of the MRC complex interventions framework.^12^

Given the overall research aim, we will consider: (1) understanding contextual factors relevant for intervention development; (2) understanding factors relevant to building programme theory and its refinement; (3) understanding key uncertainties; (4) charting information relevant for intervention refinement and (5) understanding relevant economic considerations that need to be considered for intervention development. Furthermore, we will use the Non-adoption, Abandonment, Scale-up, Spread and Sustainability framework (NASSS)^27^ framework to understand the successful implementation of the oncology fear of recurrence service including any barriers and facilitators. The flexibility afforded by also taking an inductive approach in addition to a theory driven one means we can uncover content relevant to intervention development which we may not expect in advance.

Following transcription, we will adhere to the following steps recommended by Gale:^25^

1. Familiarisation: once the first few interviews have been transcribed verbatim, SA will read the transcripts several times. They will make notes on potential themes at this stage.
2. Coding: SA will read the first five transcripts line by line and apply a code describing why each section is important. As described above, deductive and inductive coding strategy will be used, with deductive codes based on the NASSS^27^ framework (to understand implementation) and MRC complex interventions framework^12^ (to guide intervention development).
3. Developing a working analytical framework: SA will compare codes and agree on a set of deductive and inductive codes to use for coding subsequent transcripts. This framework will be reviewed by PPI contributors and other members of the research team.
4. Applying the analytical framework: the SA will systematically apply the working analytical framework to subsequent transcripts. They will also revisit previous transcripts to ensure that coding is consistent with the agreed analytical framework. The analytical framework will be updated where necessary with changes discussed with the research team including PPI colleagues.
5. Charting data into the framework matrix: the research team will summarise the data in a framework matrix. The framework matrix contains one row per participant and one column per code, with codes grouped into provisional themes and subthemes.
6. Interpreting the data: once all transcripts have been coded and charted, SA researcher will construct an overall interpretation of the data. This will result in the construction of a final set of themes which will be co-analysed with PPI colleagues.

#### Study 1—network analysis

The social network data will be analysed descriptively, looking at characteristics of (1) ego (participant), (2) alters (people the participant has interacted with in the past 3 months) and (3) network structural characteristics. We will follow the method used by Degnan *et al*^16^ and also gather information on who participants would feel comfortable discussing fear of relapse with.

- Density: the proportion of network members who know one another independent of the patient (‘ego’). This will be calculated by dividing the number of actual connections with the number of possible connections in the social network. Density ranges from 0 to 1, with higher scores indicating higher densities.
- Isolates: the number of people who are connected to the participant but are not connected to other people in the participant’s social network.
- Homophily: the extent to which participants ‘cluster’ or form relationships with people who are similar vs dissimilar to themselves (such as a shared psychosis diagnosis, gender and ethnicity). We will also report descriptive statistics for the whole sample alters showing gender, ethnicity, age and whether they are family, friends, clinicians or people who also have experience of psychosis.
- The percentage of people in a network that people discuss fear of relapse with or feel comfortable doing so with.

The information from the social network procedure will then be analysed in light of the framework analysis procedure as outlined above to give insight into how participants understand and interpret their social networks. We will also identify if there are certain characteristics that are associated with being supportive, or unsupportive in relation to fear of relapse. For example, are participants more comfortable talking about fear of relapse with people who also have experience of psychosis? Do participants find social same or different gender contacts more unhelpful?

#### Study 3

For Study 3, we will follow the framework analysis procedure outlined for Study 1 and Study 2, but will incorporate researcher summaries of the design activities (documented in a reflective log) and pictorial content such as scans of any notes that have been taken or images of participant-generated ideas rather than interview transcripts. The MoSCoW framework^28^ represents four categories of prioritisation: **m**ust-have, **s**hould-have, **co**uld-have and **w**on't-have, which will be used to understand the data in this work package.

#### Study 4

For study 4, we will follow the framework analysis procedure outlined for Study 1 and Study 2, but we will use the COM-B^29^ model as the framework for this stage. This method will allow researchers to come to a common understanding of expectations about capabilities, opportunities and motivations (COM) that participants foresee and any relevant barriers and facilitators that they imagine might be relevant for their proposed behavioural (B) engagement with the intervention including interactions with context and system fit.

#### All studies

The credibility of all analyses will be checked using reflective discussions within the research team. Additionally, further discussions may be held with the independent study steering committee and PPI colleagues.

### Ethics and dissemination

A key part of this project is developing an acceptable intervention for fear of relapse in psychosis which may have the potential to cause distress by reminding people of difficult past experiences and this was described when applying for ethical approval. This research is being conducted in NHS and community settings. The study received ethical approval from East Midlands—Nottingham 2 Research Ethics Committee (24/EM/0124).

### Dissemination

This project aims to develop an implementable low-intensity intervention for fear of relapse in psychosis. We will work with our PPI colleagues to think about how to best disseminate the findings beyond traditional methods such as publishing in peer-reviewed journals.

## Discussion

There are currently no low-intensity interventions for fear of relapse in schizophrenia. This project aims to generate knowledge about a potential novel treatment for this clinical need by using both a co-design approach and translational learning from a successful fear of recurrence intervention in oncology. Furthermore, there is limited primary qualitative research which focuses on the experiences of people who experience fear of recurrence in schizophrenia or the staff who support them.^30^ Therefore, this project will inform broader approaches to understanding the problems experienced by people diagnosed with schizophrenia and working with them to identify potentially helpful solutions.^31^

### Limitations

The proposed research has several methodological limitations that warrant consideration. First, due to resource limitations, we have limited participation to people fluent in English and who are based in a single geographic location which will introduce bias into overall participation. With these anticipated limitations in mind, we intend that this study will work in partnership with people impacted by psychosis and produce a low-intensity intervention which can be tested in later stages.

## supplementary material

10.1136/bmjopen-2024-090566 online supplemental file 1

10.1136/bmjopen-2024-090566 online supplemental file 2

10.1136/bmjopen-2024-090566 online supplemental file 3

10.1136/bmjopen-2024-090566 online supplemental file 4

10.1136/bmjopen-2024-090566 online supplemental file 5

10.1136/bmjopen-2024-090566 online supplemental file 6

10.1136/bmjopen-2024-090566 online supplemental file 7

10.1136/bmjopen-2024-090566 online supplemental file 8
