# Supplementary material for: Co-designing a low-intensity psychological therapy for fear of recurrence in psychosis using translational learning from fear of recurrence in oncology: protocol for intervention development for future testing in a feasibility study
Source: BMJ Open. 2024 Dec 27;14(12):e090566. doi: 10.1136/bmjopen-2024-090566 (PMC11683982; doi:10.1136/bmjopen-2024-090566)
Supplement: online supplemental file 1 [file bmjopen-14-12-s001.pdf]

Topic guide (Patients) Version 1.0 25/03/2024

Study title: Development, acceptability, feasibility and preliminary outcome signals for a coproduced intervention targeting fear of relapse in people with schizophrenia (INDIGO)

Work Package: A mixed-methods study of patient and staff views on developing support for people who experience fear of relapse.

What	Questions	Prompts	Notes
**Introductions and consent.**	Informed consent<br>- Anonymised transcripts and social network data; places, people, any identifiable information will be removed.<br>- Questions on experiences of fear of relapse, understanding social networks and who people discuss fear of relapse with, and what researchers could do to improve this.<br>- Remind people they do not need to answer anything they do not want to.<br>- Highlight we are interested in all experiences – good and bad.	- Any questions?	• Welcome and introductions - thank person for interest.<br>• Purpose of the interview (find out about fear of relapse and think about what might help)<br>• Confidentiality and its limits<br>• Expected timings/ breaks<br>• The digital recorder and its functioning<br>• Network canvas and its function – brief demo.<br>• Any questions? Any concerns?<br>• Informed consent<br>• Demographics form.<br>• FoRSE questionnaire

	- That we will start with demographics and a short questionnaire designed to ask questions about fear of relapse		
**Opening questions**	• How did you find filling out the questionnaire?<br>• How long have you experienced psychosis for?<br>• Sometimes people have difficult experiences of psychosis which can be called relapse, does this feel an ok term for you?<br>• What does the world relapse (or preferred term) mean for you?	• *Can you tell me more about that?*<br>• *Did this involve hospital? Crisis Team?*<br>• *What happened?*<br>- *How many times has this happened?*<br>- *How are you finding talking about this with me today?*	*To build rapport, on top of previous engagement in set up, find out anything that might make this more comfortable and start finding out about the topic. Find out about background – get demographics first and then lean into experiences of psychosis first in a structured way via questionnaire, then using more open ended questions.*
**Fear of Relapse**	• Does the term fear of relapse make sense to you? What does it mean for you?<br>• What comes to mind when you are thinking about relapse?<br>• How long have you experienced fear of relapse for?	• *Can you tell me more about that?*<br>• *What are you worried might happen?*	*Bring up fear of relapse, remind people to speak in as much detail as they feel comfortable with.*<br><br>*Normalise (if needed) that this is an understandable response to difficult experiences.*

**Fear of Relapse in daily life**	• How do you notice fear of relapse?<br>• What does fear of relapse feel like?<br>• What goes through your mind?<br>• Does anything bring up fear of relapse (e.g. places / people)?<br>• What do you do when feeling like that?<br>• Does anything help?	• *Why is that?*<br>• *Any changes in thinking, feelings in your body, memories?*<br>• *What sorts of things help? Could you tell me more please.*	*Highlight we are moving towards thinking more about impact upon daily life*
	**Social Networking Task – See Appendix C.**		
**Developing Supports**	• What are you thinking looking at your network?<br>• What support would you like to see for fear of relapse?<br>• What do you think might get in the way of providing support?<br>• What would make things better for people experiencing fear of relapse?	-	
**Ending the Interview**	• How are you feeling looking at your social network?<br>• Is there anything else you would like to tell me?<br>• How have you found the interview?<br>• How have you found the social network mapping task?<br>• Would you like a copy of the results?	- What's going through your mind?	Thank participant for their time<br><br>*To lower the intensity. Orientate person to present and future plans for post interview.*
