# Supplementary material for: Co-designing a low-intensity psychological therapy for fear of recurrence in psychosis using translational learning from fear of recurrence in oncology: protocol for intervention development for future testing in a feasibility study
Source: BMJ Open. 2024 Dec 27;14(12):e090566. doi: 10.1136/bmjopen-2024-090566 (PMC11683982; doi:10.1136/bmjopen-2024-090566)
Supplement: online supplemental file 2 [file bmjopen-14-12-s002.pdf]

Topic guide (Staff) Version 1.0 25/03/2024

Staff

Study title: Development, acceptability, feasibility and preliminary outcome signals for a coproduced intervention targeting fear of relapse in people with schizophrenia (INDIGO)

Work Package: A mixed-methods study of patient and staff views on developing support for people who experience fear of relapse.

What	Questions	Prompts	Notes
**Introductions and consent.**	Informed consent<br>- Anonymized transcripts; places, people, any identifiable information are removed.<br>- Questions on experiences of fear of recurrence in your clinical practice.<br>- Remind people they do not need to answer anything they do not want to.<br>- Highlight we are interested in all experiences<br>- Thank participant for giving up their time with their busy clinical work.	- Any questions?	• Welcome and introductions<br>• Purpose of the interview<br>• Confidentiality and its limits<br>• Expected timings/ breaks<br>• Any questions? Any concerns?<br>• The digital recorder and its functioning<br>• Informed consent and Privacy notice.<br>• Demographics form.

**Opening questions**	- What does relapse in psychosis look like?<br>- Is there anything specific to your profession relevant for relapse prevention?<br>- What relapse prevention work does your service do?<br>- What relapse prevention focus does the board take?<br>- What do patient's supporters do?<br>- What about patients themselves?<br>- Does anything get in the way of relapse prevention?	- *Can you tell me more about that?*	*To build rapport*
**Context**	- What are the consequences of relapse?<br>- For patients?<br>- For Families<br>- For Staff<br>- For health services?	- *In what ways?*	
**Fear of Relapse – mapping out**	- What do you think of the term fear of relapse?<br>- What do patients think of relapse?<br>- What impact do you think fear of relapse has (if any) on patients?<br>- How would you recongise fear of relapse in clinical practice?<br>- What impact do you think fear of relapse has on staff?	*.Could you tell me more about that please?*	

**Service Design**	- What do you think about treating worries like fear of relapse in psychosis using nonpharmacological approaches?<br>- What would be the positives?<br>- Any negatives?<br>- Who would be best placed to deliver this?<br>- When would be the best time to work with patients experiencing fear of relapse?	*- Could you tell me more about that please?*<br><br>*- what makes you think that?*	
**Ending the Interview**	- Is there anything else you would like to tell me?<br>- How have you found the interview?<br>- Is there anything we can do to improve the experience for other people?<br>- Would you like a copy of the results?		Thank participant for their time
