# Supplementary material for: Co-designing a low-intensity psychological therapy for fear of recurrence in psychosis using translational learning from fear of recurrence in oncology: protocol for intervention development for future testing in a feasibility study
Source: BMJ Open. 2024 Dec 27;14(12):e090566. doi: 10.1136/bmjopen-2024-090566 (PMC11683982; doi:10.1136/bmjopen-2024-090566)
Supplement: online supplemental file 3 [file bmjopen-14-12-s003.pdf]

Study title: Development, acceptability, feasibility and preliminary outcome signals for a coproduced intervention targeting fear of relapse in people with schizophrenia (INDIGO)

Work Package: A mixed-methods study of patient and staff views on developing support for people who experience fear of relapse.

Quantitative Social Network Measures and Concentric Circles Diagram Task

Prompt:

Thank you for discussing your experiences of fear of relapse with me, I'd like to move on and ask questions about your social network. We want to find out who you have been in touch with in the past three months. We will use the Network Canvas software. Just a reminder, everything you say will remain in confidence unless you say anything that makes me worry that you or someone else is at risk of harm, or you report poor practice.

1. Name Generator Task.

We will first try to list all people that you have been in touch with in the last **three months** – adapted from Gatej (2022).

- Who do you talk to about important things? (ie., significant persons networks)
- Who do you hang out with? These can be people you met/are speaking to face-to-face, but also online. (ie., social media)
- Who helps you with tasks like shopping etc (if relevant)? (ie., exchange/support networks)

- Thinking of more specific roles, name any other people in these networks (ie., role-focused networks):

- Parent/carers, family members such as siblings / cousins, and partners ?
- Friends and acquaintances?
- Clinicians?
- (Voluntary) Work colleagues?
- Neighbours, or anyone else that doesn't fit in the other groups?
- What people have you fallen out with or have been unhelpful with managing your experience of psychosis?

Thank you for creating this list with me, how are you feeling? Is anyone missed out?

2. Name Interpreter Task (Adapted from Gatej 2022).

I'd now like to ask you a bit of information about the people you have listed, such as their gender, age, ethnicity, how you know them, and how comfortable you feel discussing fear of relapse. We know you might not be 100% sure about age or ethnicity, in these cases your best guess is absolutely fine. It is also totally ok to say you do not know.

ID	Role	Gender	Age	Ethnicity	How long known them?	How often are you in touch with them?	Personal experience of psychosis?	Personal experience of other mental health problems (not psychosis)?	Comfort discussing fear of relapse (0 not at all to 10 most comfortable)	Comfort discussing general mental health (0 not at all to 10 most comfortable)	Comfort discussing physical health 0 not at all to 10 most comfortable)

									Prompt: What makes this comfortable / uncomfortable?		
1…											
2….											

Thank you very much. I now have more of a sense of who is in your social network.

3. Concentric Circles Task

Key opening statement:

On this page, I have a series of four circles one within the other. Imagine you are at the centre of this.

What we are going to do is place people in your life in these circles. This will give us a picture of the closeness you feel with them. The people in the circles closest to the middle are those people with whom you feel closest to/ who are most involved in your life. People in the larger circles further away are important to you but to whom you may not feel as close to.

I will then ask you to map out who knows you in your social network. We will ask this by asking if they are ever in touch with each other (including in person, online or via the phone) without you having to be there.

(Adapted from Young & Mufson, 2016 and Gatej 2022)

End:

Thank you for completing the social networking task. How did you find it? Would you like a break?

I would now like to ask some questions on your views about what might help people experience fear of relapse.
