# Supplementary material for: Co-designing a low-intensity psychological therapy for fear of recurrence in psychosis using translational learning from fear of recurrence in oncology: protocol for intervention development for future testing in a feasibility study
Source: BMJ Open. 2024 Dec 27;14(12):e090566. doi: 10.1136/bmjopen-2024-090566 (PMC11683982; doi:10.1136/bmjopen-2024-090566)
Supplement: online supplemental file 4 [file bmjopen-14-12-s004.pdf]

Topic guide_Version 1.0 25/03/2024

Service Users

Study title: Development, acceptability, feasibility and preliminary outcome signals for a coproduced intervention targeting fear of relapse in people with schizophrenia (INDIGO)

Work Package: A qualitative study of service user and staff experiences of the fear of recurrence service provided by Beatson Cancer Charity.

What	Questions	Prompts	Notes
**Introductions and consent.**	Informed consent and to highlight the following:<br>- Anonymized transcripts will be created; places, people, and any identifiable information.<br>- That there will be questions on experiences of fear of recurrence, your experiences of the Beatson Cancer Charity Fear of Recurrence Service including what was good, what was less good and ideas for improvement.<br>- Your views on any changes in your life, behavior, thinking or feelings that you	- Any questions?	• Welcome and introductions<br>• Purpose of the interview (find out about experiences of the Fear of Recurrence Service)<br>• Confidentiality and its limits<br>• Expected timings/ breaks<br>• Any questions? Any concerns?<br>• The digital recorder and its functioning<br>• Informed consent and Privacy notice.<br>• Demographics form.

	have noticed since being part of the group.<br>- A reminder for participants that they do not need to answer anything they do not want to.<br>- Highlight we are interested in all experiences – good and bad.<br>- That SA is an independent researcher and not a clinician.		
**Opening questions**	- How did you first hear about the Fear of Recurrence Group?<br>- How long had you been experiencing issues related to fear of recurrence<br>- How did you feel about getting involved in the group?<br>- How have you found the group overall?	*Could you tell me more about that please?*	*To build rapport, to get a general "temperature check" of overall experiences.*
**Experiences of the Group**	- Did you attend the group online or in-person?<br>- What was it like discussing fear of recurrence in the group?<br>- What else did you discuss in the group?<br>- How did you feel in the group?<br>- What is your strongest memory of the group?	• *Can you tell me more about that please?*<br>• *Why is that?*	*To discover overall experiences, to highlight Contextual factors such as the group being online or in person.*

**Therapist / Facilitator**	- What was your experience with the facilitators like?<br>- Have you had a talking therapy or attended a therapeutic group before for anything else? How did this compare?<br>- How did the group compare with speaking about fear of recurrence with others in your life?	• *Why is that?*<br>• *Did anything about your experience with them stand out?*	*To find out more about potential active ingredients that create change in low-intensity interventions. And build programme theory.*
Implementation	- What encouraged you to attend sessions?<br>- Were there ever times you struggled to attend sessions?<br>- If person dropped out, explore reasons for this (sensitively)	*Why is that?*<br><br>*Could you please tell me a wee bit more about that.*<br><br>*It sounds like you left the group, would it be alright to find out a bit more about why this was? I am interested in your experience.*	To understand implementation behaviours (engagement) from service users' point of view.
**Lessons Learned**	- What would you say to someone experiencing fear of recurrence who is thinking about taking part?<br>- Have you noticed any changes in your thinking or how you feelfrom taking part in the group?<br>- What do you think has worked well in the group?	*Could you tell me more about that please?*	*To expand upon implementation and explore what (if anything) has been sustained.*

	- Do you feel taking part in this group has reduced or had an impact on fear of recurrence ?<br>- What do you think has worked less well in the group?<br>- What would an ideal service for Fear of Recurrence look like?		
**Ending the Interview**	- Is there anything else you would like to tell me?<br>- How have you found the interview?<br>- Is there anything we can do to improve the experience for other people?<br>- Would you like a copy of the results?		Thank participant for their time and sort payment.<br><br>*To lower the intensity. Orientate person to present and future plans for post interview.*
