# Supplementary material for: Co-designing a low-intensity psychological therapy for fear of recurrence in psychosis using translational learning from fear of recurrence in oncology: protocol for intervention development for future testing in a feasibility study
Source: BMJ Open. 2024 Dec 27;14(12):e090566. doi: 10.1136/bmjopen-2024-090566 (PMC11683982; doi:10.1136/bmjopen-2024-090566)
Supplement: online supplemental file 5 [file bmjopen-14-12-s005.pdf]

Topic guide_Version 1.0 25/03/2024

Staff

Study title: Development, acceptability, feasibility and preliminary outcome signals for a coproduced intervention targeting fear of relapse in people with schizophrenia (INDIGO)

Work Package: A qualitative study of service user and staff experiences of the fear of recurrence service provided by Beatson Cancer Charity.

What	Questions	Prompts	Notes
**Introductions and consent.**	Informed consent<br>- Anonymized transcripts; places, people, any identifiable information are removed.	- Any questions?	• Welcome and introductions<br>• Purpose of the interview<br>• Confidentiality and its limits<br>• Expected timings/ breaks

	- Questions on experiences of fear of recurrence in your clinical practice.<br>- Remind people they do not need to answer anything they do not want to.<br>- Highlight we are interested in all experiences.<br>- Thank participant for giving up their time with their busy clinical work.		• Any questions? Any concerns?<br>• The digital recorder and its functioning<br>• Informed consent and Privacy notice.<br>• Demographics form.
**Opening questions**	- What's your experience of fear of recurrence in patients? Do you see it often? Do you think patients feel they can bring it up?<br>- How would you best describe fear of recurrence ?<br>- How do you tend to recognise fear of recurrence in your patients? What sort of things do people say? What sort of things do people do?<br>- Is there a typical presentation?<br>- Are there any unusual ways fear of recurrence presents?	- *Can you tell me more about that?*	*To build rapport and find out person's view on fear of recurrence in relation to ther clinical work.*

NHS Greater Glasgow and Clyde Indigo University of Glasgow

	- Do you ask people about their fear of recurrence? If so, how do people find being asked about experiences fear of recurrence in clinical encounters?		
**Clinical Pathways**	- How do you decide fear of recurrence is impacting someone's life enough to need intervention or support?<br>- Is there anything that makes you feel support for fear of recurrence is not needed at this time for someone?<br>- Is there anything about your specific profession relevant for discussing fear of recurrence with patients?<br>- What do you think helps people with fear of recurrence?	- *Are there any differences between different types of cancer / demographics that might be relecant?*	*To think about optimal treatment pathways and how decisions about referring to services is made.*

**Knowledge of local service provision**	- What is available locally for fear of recurrence?<br><br>**If they know about the fear of recurrance service provided by Beatson Cancer Charity.**<br><br>- If they know about Fear of Recurrence Service - How did you first learn about the fear of recurrence service?<br>- What do you think about the fear of recurrence service?<br>- What helps you decide to refer to the fear of recurrence service?<br>- What might put you off referring someone to the fear of recurrence service?<br><br>**If they do not know about the fear of recurrance service -**	*Could you tell me more about that please?*	

	- What information would you like to know about the fear of recurrance service?		
**Ending the Interview**	- Is there anything else you would like to tell me?<br>- How have you found the interview?<br>- Is there anything we can do to improve the experience for other people?<br>- Would you like a copy of the results?		Thank participant for their time
