# Supplementary material for: Co-designing a low-intensity psychological therapy for fear of recurrence in psychosis using translational learning from fear of recurrence in oncology: protocol for intervention development for future testing in a feasibility study
Source: BMJ Open. 2024 Dec 27;14(12):e090566. doi: 10.1136/bmjopen-2024-090566 (PMC11683982; doi:10.1136/bmjopen-2024-090566)
Supplement: online supplemental file 7 [file bmjopen-14-12-s007.pdf]

Workshop Session (Staff) 1
Group Design Session Set Up 1
Room Booking 2
Arrival 2
Brief Introduction 2
Icebreaker Task. 2
Group Rule Session 2
Run through of the session. 2
Introduction to the Problem. 2
Cool Down Activity 4
Ending 4
One-on-One Design Session 4
Introduction to the Problem. 4
Cool Down Activity 5

Study title: Development, acceptability, feasibility and preliminary outcome signals for a coproduced intervention targeting fear of relapse in people with schizophrenia (INDIGO)

Work Package Title: Designing a low-intensity intervention for fear of relapse in psychosis using co-design workshops.

Workshop Session (Staff)

If participants indicate they would like to take part in a group workshop session, the following set up procedure will be used.

Group Design Session Set Up

If participants indicate they would like to take part in a group workshop session.

Following discussion of the PIS and having the opportunity to ask any questions and being advised on issues key to informed consent such as it being voluntary and right of withdrawal, staff participants will be invited to consent formally into the study.

Consent will be taken before workshop participation. Attendees will be given the location, time and date and this will be sent via an appointment letter.

Room Booking

A main room will be booked on the ground floor of a University Building. Additionally, a "quiet room" will be booked nearby the space which will be for the exclusive use of workshop attendees.

Arrival

A desk will be manned by two trained researchers (wearing name badges) to welcome staff attendees to the workshop, they will be given printed information with a plan for the day including expected timings and a name badge. The researcher will formally introduce themselves and answer any questions, and guide the participants into the main room.

Brief Introduction

Once everyone is seated and comfortable in the main room, the researchers will introduce themselves more formally and begin the Icebreaker Task

Icebreaker Task.

In line with good co-design workshop practice, we will begin the day with an ice breaker task. We will use a "four quadrants" approach which splits the room up into four sections (indicated by a printout on each wall). Participants will be asked what are you looking forward to, with four options:

1) Trying something new
2) Sharing my expertise in psychosis care
3) Developing a new intervention
4) Other (share if you feel comfortable)

Workshop participants will be invited to stand next to the area that suits them best.

Group Rule Session

Following completion of the Icebreaker Task, participants will then be invited to take part in a brainstorming session to produce group rules to guide the day.

Run through of the session.

Following the establishment of Group Rules, participants will be provided with a short presentation that includes an overview of the day. This will match the printed schedule provided when they came in.

Introduction to the Problem.

The researchers will give a short presentation which orientates the participants to the problem of fear of relapse, and updates on the findings from WP1.1. This is a knowledge sharing activity which explains what insights we have already received from mental health staff and patients who have participated in WP1. Additionally, we will share WP1.2 learning which comes from our NHS and third oncology colleagues, as well as the direct views of service users who have accessed the fear of recurrence service.

This presentation will also highlight that the scope of the project is to develop a low intensity intervention and will describe what this involves.

The presentation will highlight that there is no right or wrong, and that we are very interested in finding out why people think what they do.

The workshop activities are designed to gather stakeholder views on three key thematic areas: 1) Intervention Content, 2) Intervention Delivery, and 3) Journey Mapping (to understand when an intervention should be offered).

Intervention Content

Aim: To discover what content should be in a low-intensity intervention for Fear of Relapse

1) Researchers present themes from WP1 as tags on the wall.
2) Blank tags are provided so people can write down missing things,
3) Participants rate themes as “keep,” “lose,” or “change” using colour-coded post-it notes. These will be indicated by sections on the wall.

Intervention Delivery

1) Researchers will present A4 paper sheets with blank therapy module mock-ups
2) Participants formulate ideas or write descriptions of how the information could be presented during therapy sessions.

Journey Mapping

Participants will be invited to map out what a current user journey looks like for people who experience fear of relapse.

1) Researchers present an editable map with locations people with psychosis may find themselves: first episode, hospital, CPN appointments, and time between appointments.
2) Participants can indicate where they think people would want to engage in brief therapy.

Cool Down Activity

This will orientate participants to the end of the co-design workshops and returning to their day. We will use a whiteboard and invite participants to share what they have learned from today.

Participants will be given the debrief letter.

Ending

Provision of lunch. Lunch bags will be available for people who want to grab food and go, or who do not feel comfortable eating around other people.

The researchers will be available for an informal chat.

One-on-One Design Session

Set Up

Following discussion of the PIS and having the opportunity to ask any questions and being advised on issues key to informed consent such as it being voluntary and right of withrawl staff will be invited to consent formally into the study and an appointment will be made to do this which can be either in person or online depending on risk assessment and availability of rooms.

Run through of the session.

Introduction to the Problem.

The researchers will give a short presentation which orientates the participants to the problem of fear of relapse, and updates on the findings from WP1.1. This is a knowledge sharing activity which explains what insights we have already received from mental health staff and patients who have participated in WP1. Additionally, we will share WP1.2 learning which comes from our NHS and third oncology colleagues, as well as the direct views of service users who have accessed the fear of recurrence service.

This presentation will also highlight that the scope of the project is to develop a low intensity intervention and will describe what this involves.

The presentation will highlight that there is no right or wrong, and that we are very interested in finding out why people think what they do.

The workshop activities are designed to gather stakeholder views on three key thematic areas: 1) Intervention Content, 2) Intervention Delivery, and 3) Journey Mapping (to understand when an intervention should be offered).

Intervention Content

Aim: To discover what content should be in a low-intensity intervention for Fear of Relapse

1) The researcher will present themes from WP1 as either tags (for in person meetings) or as electronic tags on a Microsoft Powerpoint using the screen sharing function (for online meetings)
2) Blank tags will be provided so people can write down missing things.
3) Participants will be welcomed to rate the themes as “keep,” “lose,” or “change”.

Intervention Delivery

1) The researcher will present A4 paper sheets with blank therapy module mock-ups (for in person session) or as a Powerpoint Presentation using the screen share function (for online meetings)
2) Participants will be invited to formulate ideas or write descriptions of how the information could be presented during therapy sessions.

Journey Mapping

Participants will be invited to map out what a current user journey looks like for people who experience fear of relapse.

1) The researcher will present an editable map with locations people with psychosis may find themselves: first episode, hospital, CPN appointments, and time between appointments.
2) Participants can indicate where they think people would want to engage in brief therapy.

Cool Down Activity

This will orientate participants to the end of the co-design workshops and returning to their day. The researcher will summarise the workshop and invite participants to share what they have learned from today.

Participants will be given the debrief letter.
