# Supplementary material for: Co-designing a low-intensity psychological therapy for fear of recurrence in psychosis using translational learning from fear of recurrence in oncology: protocol for intervention development for future testing in a feasibility study
Source: BMJ Open. 2024 Dec 27;14(12):e090566. doi: 10.1136/bmjopen-2024-090566 (PMC11683982; doi:10.1136/bmjopen-2024-090566)
Supplement: online supplemental file 8 [file bmjopen-14-12-s008.pdf]

Topic guide Version 1.0 25/03/2024

Study title: Development, acceptability, feasibility and preliminary outcome signals for a coproduced intervention targeting fear of relapse in people with schizophrenia (INDIGO)

Work Package Title: Usability testing of an intervention designed to support people who experience fear of relapse: a qualitative think aloud study.

What	Questions	Prompts	Notes
**Introductions and consent.**	Informed consent and to highlight the following:<br>- That there will be questions and activities to better understand your recent mental and cognitive health.<br>- Following this, we will present some information about a newly developed talking therapy to support people experiencing fear of relapse. We will not be doing therapy today, but are interested to find out what you think about this idea. We will ask you to "think aloud" as you are interacting with the material.<br>- Anonymized transcripts will be created; places, people, and any identifiable information.<br>- A reminder for participants that they do not need to answer anything they do	- Any questions?	• Welcome and introductions<br>• Purpose of the session (we want to make sure hear the views of people who experience psychosis, and we want to make sure people who may have struggles with things like concentrating or hearing voices are represented. We will ask some standard questions to help us confirm we are gathering a range of perspectives.)<br>• Confidentiality and its limits<br>• Expected timings/ breaks<br>• Any questions? Any concerns?<br>• The digital recorder and its functioning<br>• Informed consent.<br>• Demographics form.

	not want to. We can have as many breaks as you need.<br>- Highlight we are interested in all views of this proposed project– good and bad.		
**Opening Procedure**	Completing demographics form.<br><br>Completing BACS and CGI.	*how are you finding going through these questions with me today?*	
**Introduction to intervention and thinking aloud.**	Script:<br><br>I am going to ask you to think aloud as you go through the outline of an intervention that has been developed to support people who worry about relapse. The outline is printed out onto pages that you can flick through. Thinking aloud means I am interested in finding out everything that you think as you go through it in as much detail as you are comfortable sharing with me. Please do not worry about what to say or spend time trying to think about what to say, I'm just interested in finding out what comes to mind for you as you go through the material. You might need some time to read the material and think what to say, that is totally ok – we can go at your own pace. Do let me know if any of the material is unclear or if you have any questions.<br><br>I know it might feel unusual or even awkward to share your thoughts out loud in this way. You might not have done anything like this before. I just wanted to say I am not	• *Any questions?*	*To introduce thinking aloud, normalize any strangeness, emphasise that this is not a test, ensure people have control of what they share and are priorotising their comfort and safety.*

	here to analyse your thinking or make any judgements about you, I am just genuinely interested to find out what your views on this project. As it can be hard to just get started with thinking out loud, I have some standard questions to get you started.		
**I**magined Capabilities	- Exploring views on mode of delivery such as proposed therapeutic content such as card sort exercises or social network creation. - Exploring views on the language choice used and accessibility of materials. - Exploring views if the required level of sessions is appropriate - Exploring views on what support people may need to engage. - What else is needed?	*Why is that?* *What is going through your mind when you look at that?* *Could you tell me a bit more about that please?* *If people stop speaking, lead researcher to thank them for their time and say they are doing well and use prompts like "what are you thinking now?"*	
**Imagined** Opportunities	- Exploring views about the content of the proposed intervention such as how useful you think it will be, and its relevance. - Or if you do not think it is useful or relevant, why – and what could make more useful / relevant. - What else is needed?	*Why is that?*<br><br>*Could you please tell me a wee bit more about that.*<br><br>*If people stop speaking, lead researcher to thank them for their time and say they are doing well and use prompts like "what are you thinking now?"*	

Imagined Motivations	• Exploring views about what might make someone want to take part in this intervention<br><br>• Exploring views about what might put someone off taking part in this intervention.<br><br>• What could be done to support engagement?	*Could you tell me more about that please?*<br><br>*Why do you think that is?*<br><br>*If people stop speaking, lead researcher to thank them for their time and say they are doing well and use prompts like "what are you thinking now?"*	
**Ending the Interview**	- Is there anything else you would like to tell me?<br>- How have you found the interview?<br>- Is there anything we can do to improve the experience for other people?<br>- Would you like a copy of the results?		
