# Supplementary material for: Co-designing a low-intensity psychological therapy for fear of recurrence in psychosis using translational learning from fear of recurrence in oncology: protocol for intervention development for future testing in a feasibility study
Source: BMJ Open. 2024 Dec 27;14(12):e090566. doi: 10.1136/bmjopen-2024-090566 (PMC11683982; doi:10.1136/bmjopen-2024-090566)
Supplement: Supplementary file 9 [file bmjopen-2024-090566.reviewer_comments.pdf]

PEER REVIEW HISTORY

BMJ Open publishes all reviews undertaken for accepted manuscripts. Reviewers are asked to complete a checklist review form and are provided with free text boxes to elaborate on their assessment. These free text comments are reproduced below.

ARTICLE DETAILS

Title (Provisional)

Co-designing a low-intensity psychological therapy for fear of recurrence in psychosis using translational learning from fear of recurrence in oncology: protocol for intervention development for future testing in a feasibility study.

Authors

Allan, Stephanie; sinclair, fiona; Correia, Marta; FRAGKANDREA-NIXON, Ioanna na; Phiri, Alie; Jones, Gareth; Thomson, David; Yanga, Francis; Brown, George; McCann, Mark; Simpson, Sharon Anne Anne; Evans, Jonathan; Robb, Katie; Gumley, Andrew

---

This article was not externally reviewed at BMJ Open. Protocols that have been independently assessed prior to submission to BMJ Open are usually fast-tracked to publication on the grounds that further substantial changes will not be possible. This independent assessment will usually be external, independent review for both a substantial grant award from a non-commercial or government funder and ethics approval.
